# Diaphragm function in patients with Covid-19-related acute respiratory distress syndrome on venovenous extracorporeal membrane oxygenation

**DOI:** 10.1186/s13613-023-01179-w

**Published:** 2023-09-26

**Authors:** Melchior Gautier, Vincent Joussellin, Jacques Ropers, Lina El Houari, Alexandre Demoule, Thomas Similowski, Alain Combes, Matthieu Schmidt, Martin Dres

**Affiliations:** 1 Sorbonne Université, Institute of Cardiometabolism and Nutrition, Institut National de la Santé et de la Recherche Médicale (INSERM) Unité Mixte de Recherche (UMRS) 1166, Paris, France; 2 Service de Médecine Intensive–Réanimation, Institut de Cardiologie, Assistance Publique–Hôpitaux de Paris (APHP), Hôpital Pitié–Salpêtrière, Paris, France; 3 Groupe de Recherche Clinique 30 RESPIRE, Paris, France; 4 Sorbonne Université, INSERM, UMRS1158 Neurophysiologie Respiratoire Expérimentale et Clinique, 75005 Paris, France; 5 Département R3S (Respiration, Réanimation, Réadaptation Respiratoire, Sommeil), AP-HP, Groupe Hospitalier Universitaire APHP-Sorbonne Université, Hôpital Pitié-Salpêtrière, 75013 Paris, France; 6 Département de Santé Publique, AP-HP, Assistance Publique–Hôpitaux de Paris (APHP), Hôpital Pitié–Salpêtrière, Paris, France; 7 Assistance Publique-Hôpitaux de Paris, Pitié-Salpêtrière Hospital Medical Intensive Care Unit, 47-83 Boulevard de l’Hôpital, 75013 Paris, France

**Keywords:** Diaphragm dysfunction, Weaning, Ventilator-induced diaphragm dysfunction, ECMO, ARDS

## Abstract

**Background:**

Venovenous extracorporeal membrane oxygenation (VV ECMO) is frequently associated with deep sedation and neuromuscular blockades, that may lead to diaphragm dysfunction. However, the prevalence, risk factors, and evolution of diaphragm dysfunction in patients with VV ECMO are unknown. We hypothesized that the prevalence of diaphragm dysfunction is high and that diaphragm activity influences diaphragm function changes.

**Methods:**

Patients with acute respiratory distress syndrome (ARDS) requiring VV ECMO were included in two centers. Diaphragm function was serially assessed by measuring the tracheal pressure in response to phrenic nerve stimulation (Ptr,stim) from ECMO initiation (Day 1) until ECMO weaning. Diaphragm activity was estimated from the percentage of spontaneous breathing ventilation and by measuring the diaphragm thickening fraction (TFdi) with ultrasound.

**Results:**

Sixty-three patients were included after a median of 4 days (3–6) of invasive mechanical ventilation. Diaphragm dysfunction, defined by Ptr, stim ≤ 11 cmH_2_O, was present in 39 patients (62%) on Day 1 of ECMO. Diaphragm function did not change over the study period and was not influenced by the percentage of spontaneous breathing ventilation or the TFdi during the 1 week. Among the 63 patients enrolled in the study, 24 (38%) were still alive at the end of the study period (60 days).

**Conclusions:**

Sixty-two percent of patients undergoing ECMO for ARDS related to SARS CoV-2 infection had a diaphragm dysfunction on Day 1 of ECMO initiation. Diaphragm function remains stable over time and was not associated with the percentage of time with spontaneous breathing.

*ClinicalTrials.gov Identifier* NCT04613752 (date of registration February 15, 2021).

**Supplementary Information:**

The online version contains supplementary material available at 10.1186/s13613-023-01179-w.

## Background

Invasive mechanical ventilation aims to maintain adequate gas exchanges in acute respiratory distress syndrome (ARDS) while resting the respiratory muscles. Its current management is based on lung protective ventilation which combines tidal volume and plateau pressure reduction to limit the harmful effects of positive pressure ventilation on the alveoli, a phenomenon referred to as ventilator-induced lung injury (VILI) [1–3]. VILI is recognized to be the constellation of pulmonary consequences of mechanical ventilation that could potentially lead to an increase in the systemic inflammatory response and contribute to multiorgan failure [4]. A similar concern has also emerged about the potential adverse effects of invasive mechanical ventilation on the respiratory muscles. This entity was originally termed ventilator-induced diaphragmatic dysfunction which has been associated with prolonged duration of mechanical ventilation, difficult and prolonged weaning, and poor prognosis [5–7]. In severe ARDS refractory to conventional management, venovenous extracorporeal membrane oxygenation (VV ECMO) provides full extracorporeal blood oxygenation and carbon dioxide removal, to replace pulmonary function. To further limit the energy transmitted to the lungs by the mechanical ventilator, “ultra-lung-protective” ventilation reducing tidal ventilation, respiratory rate, and plateau and driving pressures is commonly used in combination with VV-ECMO [8]. Such a strategy requires heavy sedation which leads to a forced rest of the respiratory muscles, primarily the diaphragm, with a time-dependent dysfunction [9, 10], and atrophy [11, 12]. Additionally, prolonged time with non-invasive oxygenation strategies before ECMO may also expose these patients to excessive respiratory efforts [13] and subsequent diaphragm injury [14]. Although the influence of diaphragm dysfunction on the outcomes of invasively mechanically ventilated patients is well described [12], the diaphragmatic function of these patients with severe ARDS on ECMO has never been studied. Similarly, the impact of ultra-protective lung ventilation on the diaphragm function has never been studied. We aimed to report the prevalence, time course, and factors associated with diaphragm dysfunction in a population of severe ARDS on VV-ECMO with a particular focus on the influence of diaphragm activity resulting from spontaneous breathing on the diaphragm function changes. We hypothesized that the prevalence of diaphragm dysfunction is high and that diaphragm activity influences diaphragm function changes.

## Methods

We conducted a prospective observational study in two intensive care units of the Pitié-Salpêtrière Hospital (Assistance Publique–Hôpitaux de Paris) over 8 months between March and October 2021. This study was approved by the ethical committee (Comité de Protection des Personnes du Sud-Est 5, RCB ID: 2019-A02637-50). Written informed consent was obtained from all patients’ relatives before inclusion. The study was registered on ClinicalTrials.gov (NCT04613752) prior inclusion of the first patient and followed the STROBE reporting guidelines for observational studies.

### Patients

Inclusion criteria were: (1) patient with severe ARDS on VV ECMO for less than 24 h, (2) on pressure-controlled mechanical ventilation mode, and (3) sedated with a Richmond assessment scale (RASS) ≤ –2. Exclusion criteria were (1) age < 18 years, (2) known pregnancy, (3) contraindications to magnetic stimulation of the phrenic nerves (e.g., cardiac pacemaker or implanted defibrillator, cervical implants), and (4) expected death within 24 h.

### Protocol

The participating ICUs are regional referral ECMO centers where patients are usually retrieved from non-ECMO centers after ECMO implantation by the mobile ECMO team [15]. As soon as ECMO started, all patients were ventilated using a V500 ventilator (Dräger^®^, Lübeck, Germany) in BIPAP/APRV mode with a constant driving pressure (plateau pressure minus PEEP) of 12–14 cmH_2_O (14, 15), a PEEP > 10 cmH_2_O, respiratory rate of 10–20 breaths/min and FiO_2_ to maintain SaO_2_ > 92% [3]. On ECMO, the level of sedation was monitored four times daily by the RASS. Spontaneous breathing on BIPAP/APRV was allowed. Of note, spontaneous breathing on BIPAP/APRV is not synchronized with the pressure cycle and could be expressed by the ratio of spontaneous minute ventilation to total minute ventilation. ECMO weaning criteria followed those applied during the EOLIA trial [3].

Diaphragm function assessment, diaphragm ultrasound, and lung ultrasound were all performed together within 24 h after VV-ECMO onset (Day 1) and thereafter repeated on ECMO Day 2, 3, 7, 10, 14, 21, and 28 until ECMO weaning or death, whatever occurred first. For those patients on continuous neuromuscular blockade (atracurium), the intravenous infusion was interrupted for at least 2 h (i.e., five half-lives) before diaphragm function assessment [16].

### Diaphragm function assessment

Diaphragm function was defined as the capacity of the diaphragm to generate a negative intrathoracic pressure [17]. It was assessed by the changes in endotracheal tube pressure induced by bilateral phrenic nerve stimulation during airway occlusion (Ptr, stim). Phrenic nerve stimulation was performed by bilateral anterior magnetic stimulation, as described elsewhere [17–19]. Briefly, two figure-of-eight coils connected to a pair of Magstim^®^ 200 stimulators (The Magstim Company, Whitland, UK) were positioned immediately posterior to the sternocleidomastoid muscles at the level of the cricoid cartilage. Bilateral phrenic nerve stimulation was performed while the endotracheal tube was manually occluded, and stimulations were delivered at the maximum intensity allowed by the stimulator (100%) known to result in supramaximal diaphragm contraction. The patients were studied in a standardized semi-recumbent position during a brief disconnection of the endotracheal tube from the ventilator. While the endotracheal tube was manually occluded, bilateral anterolateral magnetic stimulation was performed. The absence of active respiratory efforts was verified by checking the absence of a drop in airway pressure signal on the laptop screen. Two operators (MG, VJ) were required to achieve both stimulation and measurements. After positioning the coils, at least three stimulations were performed. Stimulations were separated by at least 60-s to avoid superposition. Patients were not reconnected to the ventilator between stimulations. Ptr,stim was defined as the amplitude of the negative pressure wave following stimulation, taken from baseline to peak. It was measured at the proximal external end of the endotracheal tube, using a linear differential pressure transducer (MP45 ± 100 cmH_2_O, Validyne, Northridge, Calif., USA). The pressure signal was sampled and digitized at 100 Hz (MP30, Biopac Systems, Santa Barbara, Calif., USA, or Powerlab, AD Instruments, Bella Vista, Australia) for offline data analysis. The average of three measures was considered during offline and blinded analysis. A Ptr,stim ≤ 11 cmH_2_O defined diaphragm dysfunction [13, 20].

### Diaphragm ultrasound

Ultrasound was performed using two different machines in each ICU (Sparq ultrasound system, Phillips, Philips Healthcare, MA, USA, and CX50 Philips, Philips Healthcare, MA, USA) by two trained investigators (MG, VJ). Both operators had extensive experience in diaphragm ultrasound imaging and followed the same methodology to ensure the reliability of ultrasound recordings across participants. The methods to evaluate diaphragm thickness and thickening have been extensively detailed and validated elsewhere [20, 21]. Diaphragm ultrasound was conducted using a 10–15 MHz linear array transducer. Diaphragm thickness (including pleural and peritoneal membranes) was imaged on the right zone of apposition with the probe placed on the 8th to 10th intercostal space near the midaxillary line. The diaphragm was located as a muscular layer in-between two hyperechoic lines (i.e., the pleura and peritoneum), superficial to the liver. The thickening fraction (TFdi) was calculated offline as peak inspiration thickness minus end-expiratory thickness divided by end-expiratory thickness. All ultrasound measurements were repeated on at least three separate breaths and their averages were reported. The reproducibility of diaphragm ultrasound has been reported elsewhere and was not investigated in the present study [21, 22].

### Lung ultrasound

Lung ultrasound was performed by two trained investigators (MG, VJ). A 2–4 MHz probe was used to scan the whole lung on both sides. The number of B-lines was counted on a rib short-axis scan between two ribs at each intercostal space of the upper and lower parts of the anterior, lateral, and posterior regions of the left and right chest wall (a total of 12 areas). For a given region of interest, points were allocated according to the observed ultrasound pattern: the presence of lung sliding with A lines or fewer than two isolated B lines = 0, multiple, well-defined B lines = 1, multiple coalescent B lines = 2, lung consolidation = 3 [23].

### Data collection

Demographic data, severity scores, organ dysfunction–related variables, blood gas, ventilator settings, vasopressors, and inotropes doses were prospectively collected. Moreover, the proportion of spontaneous minute ventilation over the last 24 h was averaged at each diaphragm function assessment. Lastly, tracheostomy, ventilator-associated pneumonia, invasive mechanical ventilation duration, and ICU and hospital lengths of stay were also reported.

### Statistical analysis

We followed the STROBE (Strengthening the Reporting of Observational Studies in Epidemiology) recommendations for reporting cohort studies [24]. Continuous variables were summarized with their median and inter-quartile range (IQR) and categorical variables using numbers and percentages (%). Wilcoxon rank sum test and Student test were used to compare patients’ characteristics according to the presence or absence of diaphragm dysfunction at ECMO day 1 (i.e., Ptr, stim ≤ 11 cmH_2_O) for variables on a continuous scale. The Pearson test and Fisher’s exact tests were used for comparisons of categorical values. Nominal *p*-values are reported and *p*-values < 0.05 were considered statistically significant. Linear regression was used to investigate the relationships between continuous variables. The averaged percentage of spontaneous breathing ventilation on APRV and the averaged TFdi were used as surrogates of the diaphragm activity over the first 7 days. Correlation between the averaged percentage of daily spontaneous breathing ventilation within the first 7 days and Ptr,stim on Day 7 was assessed. The same analysis was done for average TFdi during the first 7 days and Ptr,stim on Day 7.

Due to the exploratory nature of our study and the lack of study references in that severe population, no formal sample size calculation was deemed necessary. We planned a convenient sample of at least 60 patients.

## Results

### Study population

**Fig. 1:**
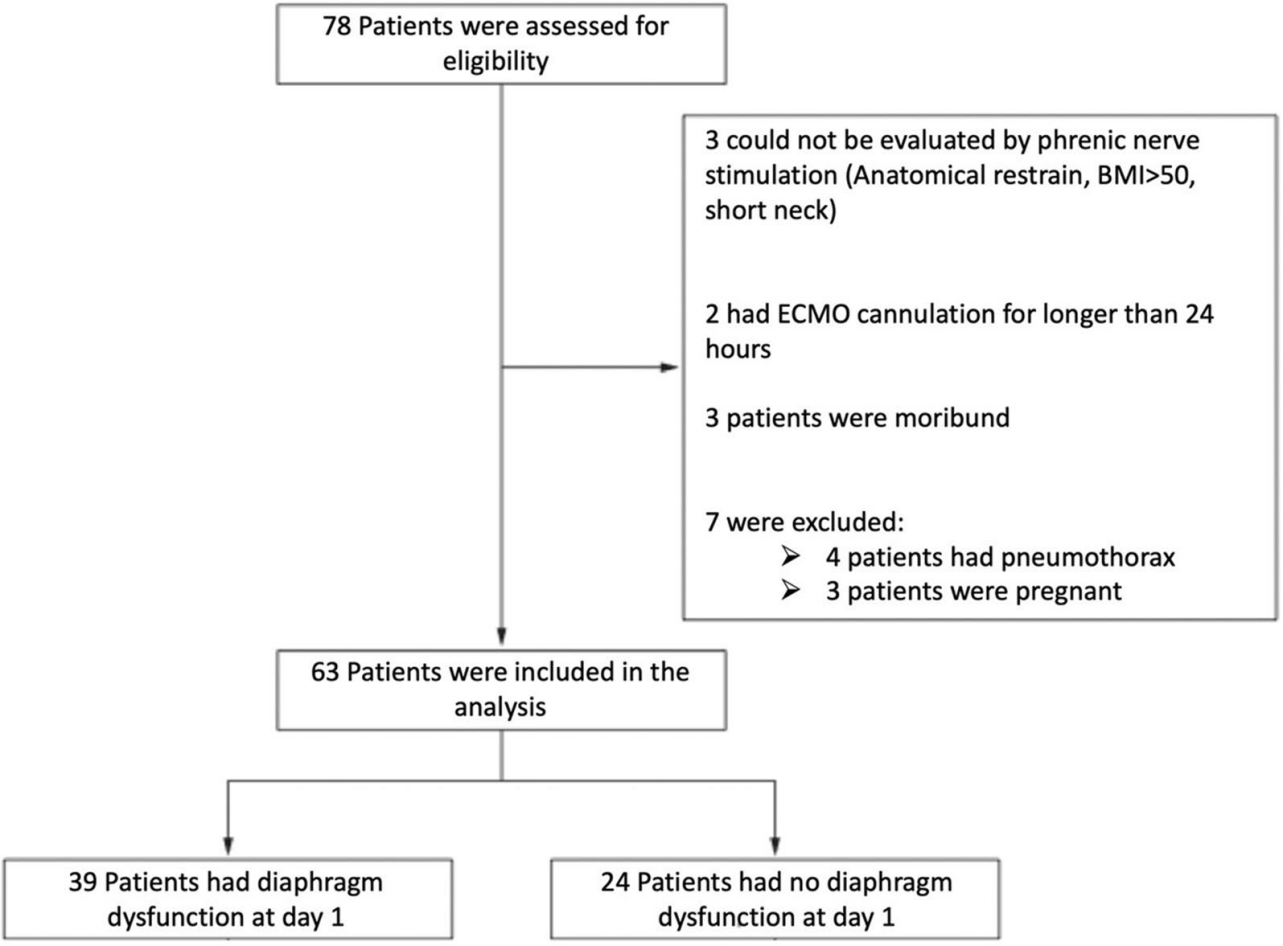
Flowchart

Between February 1st and July 31st, 2021, 78 patients with ARDS receiving VV ECMO were admitted to the two ICUs. All patients had COVID-19-related ARDS. 70 patients had all inclusion criteria. After excluding 7 patients, 63 patients were analyzed in the present study (Fig. 1). Their characteristics are reported in Table 1. Patients were predominantly male, with a median age of 53 years (42–59) and a median body mass index of 33 (29–37) kg/m^2^. Prior intubation, non-invasive ventilation, and high-flow nasal oxygen were used in 33 (54%) and 53 (85%) patients, respectively. Non-invasive respiratory support (either high-flow nasal oxygen or non-invasive ventilation) was provided for 4 (1–7) days before intubation and invasive mechanical ventilation was provided for 4 (3–6) days before ECMO. At ECMO onset, respiratory system compliance was 22 (16–25) mL/cmH_2_O, and driving pressure was 19 (16–21) cmH_2_O.

**Table 1:** Pre-ECMO patients’ characteristics according to diaphragm dysfunction at ECMO day-1

Variables	All patients (n = 63)	Diaphragm dysfunction (n = 39)	No diaphragm dysfunction (n = 24)	*P* value
Age, years, median (IQR)	53 (42–59)	55 (45–59)	47 (40–54)	0.049
Female sex, n (%)	16 (25)	10 (26)	6 (25)	0.954
Body mass index, kg/m^2^, median (IQR)	33 (29–37)	32 (29–37)	34 (28–36)	0.937
Arterial hypertension, n (%)	29 (46)	22 (56)	7 (30)	0.035
SAPS II, median (IQR)	56 (45–66)	58 (47–67)	55 (39–61)	0.191
SOFA, median (IQR)	12 (9–12)	12 (9–13)	12 (9–12)	0.540
Charlson ≥ 1, n (%)	24 (38)	15 (38)	9 (38)	0.800
High flow nasal oxygen, n (%)	53 (85)	33 (87)	20 (83)	0.724
Duration, days, median (IQR)	5 (2–7)	5 (1–7)	5 (2–7)	0.477
Non-invasive ventilation, n (%)	33 (54)	21 (57)	12 (50)	0.539
Duration, days, median (IQR)	6 (2–7)	6 (2–7)	7 (4–9)	0.438
Duration of MV before ECMO, median (IQR)	4 (3–6)	4 (3–7)	4 (2–5)	0.593
Length of stay in ICU before ECMO, days, median (IQR)	10 (6–13)	10 (6–11)	9 (6–12)	0.904
Pre-ECMO ventilator settings				
Tidal volume, ml/kg PBW, median (IQR)	6.0 (5.5–6.3)	6.0 (5.7–6.4)	5.9 (5.5–6.1)	0.144
Respiratory rate, min^−1^, median (IQR)	30 (30–33)	30 (30–32)	32 (30–34)	0.523
Driving pressure, cmH_2_O, median (IQR)	19 (16–21)	18 (16–20)	20 (20–25)	0.006
Positive end-expiratory pressure, cmH_2_O, median (IQR)	12 (10–14)	13 (12–15)	12 (10–13)	0.029
Respiratory system compliance, mL/cmH_2_O, median (IQR)	22 (16–25)	23 (16–26)	19 (12–22)	0.005
Corticosteroids, n (%)	62 (98)	38 (97)	24 (100)	0.735
Neuromuscular blocking agents, n (%)	62 (98)	37 (97)	24 (100)	0.456
Nitric oxide, n (%)	22 (35)	15 (39)	7 (30)	0.201
Prone positioning, n (%)	59 (95)	34 (89)	25 (100)	0.398
Criteria for VV-ECMO, n (%)				
PaO_2_/FiO_2_ < 50 for more than 3 h, n (%)	12(19)	9 (23)	3 (13)	0.707
PaO_2_/FiO_2_ < 80 for more than 6 h, n (%)	46 (73)	26 (67)	20 (83)	0.233
pH < 7.20 and PaCO_2_ > 70 mmHg for more than 6 h, n (%)	13 (21)	10 (26)	3 (13)	0.128

*PBW* Predicted Body Weight, *MV* Mechanical ventilation, *VAP* Ventilator-acquired pneumonia, *ICU* Intensive care unit, *SAPS II* Simplified Acute Physiology Score II, *SOFA* Sequential Organ Failure Assessment, *IQR* Inter-Quartile Range

### Diaphragm function assessment on day 1

**Fig. 2:**
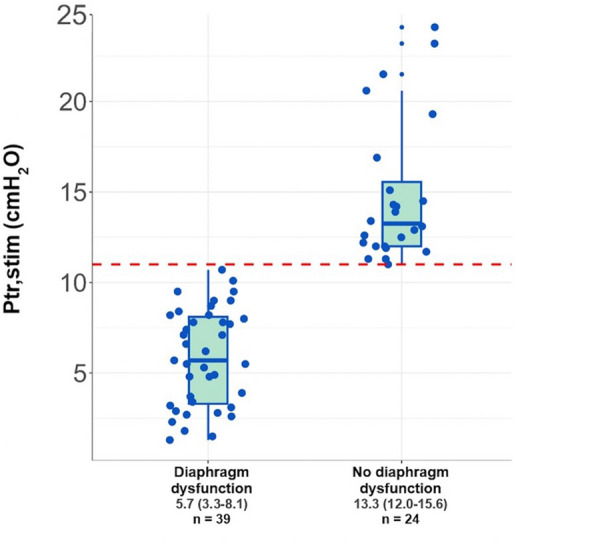
Individual Ptr,stim in patients with and without diaphragm dysfunction at ECMO Day 1. The horizontal red line represents the value of 11cmH_2_O defining the threshold value of diaphragm pressure generation in response to phrenic nerve stimulation

At ECMO day 1, Ptr,stim was 8.4 (5.1–12.5) cmH_2_O in the whole population. Thirty-nine (62%) patients had a diaphragm dysfunction on Day 1 (Ptr,stim 5.7 (3.3–8.1) cmH2O) whereas their counterparts had a Ptr,stim of 13.3 (12.0–15.6) cmH_2_O (Fig. 2). Patients with diaphragm dysfunction had more frequent hypertension and were older. Pre-ECMO non-invasive respiratory support and duration of invasive mechanical ventilation were similar between the two groups. Likewise, the dose of hypnotics, opioids, steroids, and norepinephrine was not different between groups (Table 2). By contrast, patients with diaphragm dysfunction had a significantly higher pre-ECMO PEEP level and a higher LUS score (Table 2).

**Table 2:** Patients’ characteristics at ECMO day 1 according to diaphragm dysfunction at ECMO day 1

Variables	All patients (n = 63)	Diaphragm dysfunction (n = 39)	No diaphragm dysfunction (n = 24)	*P* value
Ventilator settings				
Tidal volume, ml/kg PBW	2.8 (2.2–3.7)	2.7 (2.2–3.6)	3.0 (2.1–3.8)	0.488
Respiratory rate, /min	20 (20–23)	20 (20–22)	21 (20–23)	0.721
Driving pressure, cmH_2_O	14 (13–14)	14 (12–14)	14 (12–14)	0.566
Positive end-expiratory pressure, cmH_2_O	12 (12–14)	12 (12–14)	12 (12–14)	0.508
Respiratory system compliance, mL/cmH_2_O	14 (10–18)	13 (10–18)	16 (10–20)	0.440
Plateau pressure, cmH_2_O	26 (24–28)	26 (24–28)	26 (25–28)	0.833
Hemodynamics				
Heart rate, min^−1^	81 (64–93)	83 (65–94)	74 (63–89)	0.423
Mean arterial pressure, mmHg	73 (68–80)	73 (68–78)	74 (71–80)	0.162
Norepinephrine, µ/kg/min	0.1 (0.0–0.34)	0.1 (0.0–0.4)	0.1 (0.0–0.3)	0.658
Arterial blood lactate, mmol/L	1.8 (1.5–2.3)	1.8 (1.5–2.4)	1.8 (1.4–2.1)	0.577
Fluid balance of the last 24 h, ml	− 151 (− 709–1137)	115 (− 753–1358)	− 252 (− 675–244)	0.489
Creatinine, µmol/l	78 (62–127)	94 (67–171)	71 (49–99)	0.028
Lung ultrasound Score	26 (23–27)	26 (24–28)	25 (22–26)	0.008
White cells count, G/l	16 (12–20)	16 (12–21)	15 (10–20)	0.577
Procalcitonin, ng/l	0.6 (0.3–3.7)	1.1 (0.3–3.0)	0.6 (0.2–4.1)	0.326
Cumulated dose of sedation				
Propofol, mg/day	4800 (3600–5310)	4800 (3600–4800)	4800 (4500–6000)	0.075
Sufentanyl, µg/day	480 (360–600)	480 (360–600)	480 (360–600)	0.691
Midazolam, mg/day	480 (360–600)	480 (360–540)	480 (367–600)	0.664

*PBW* predicted body weight

### Time course of diaphragm function over the ICU stay

**Fig. 3:**
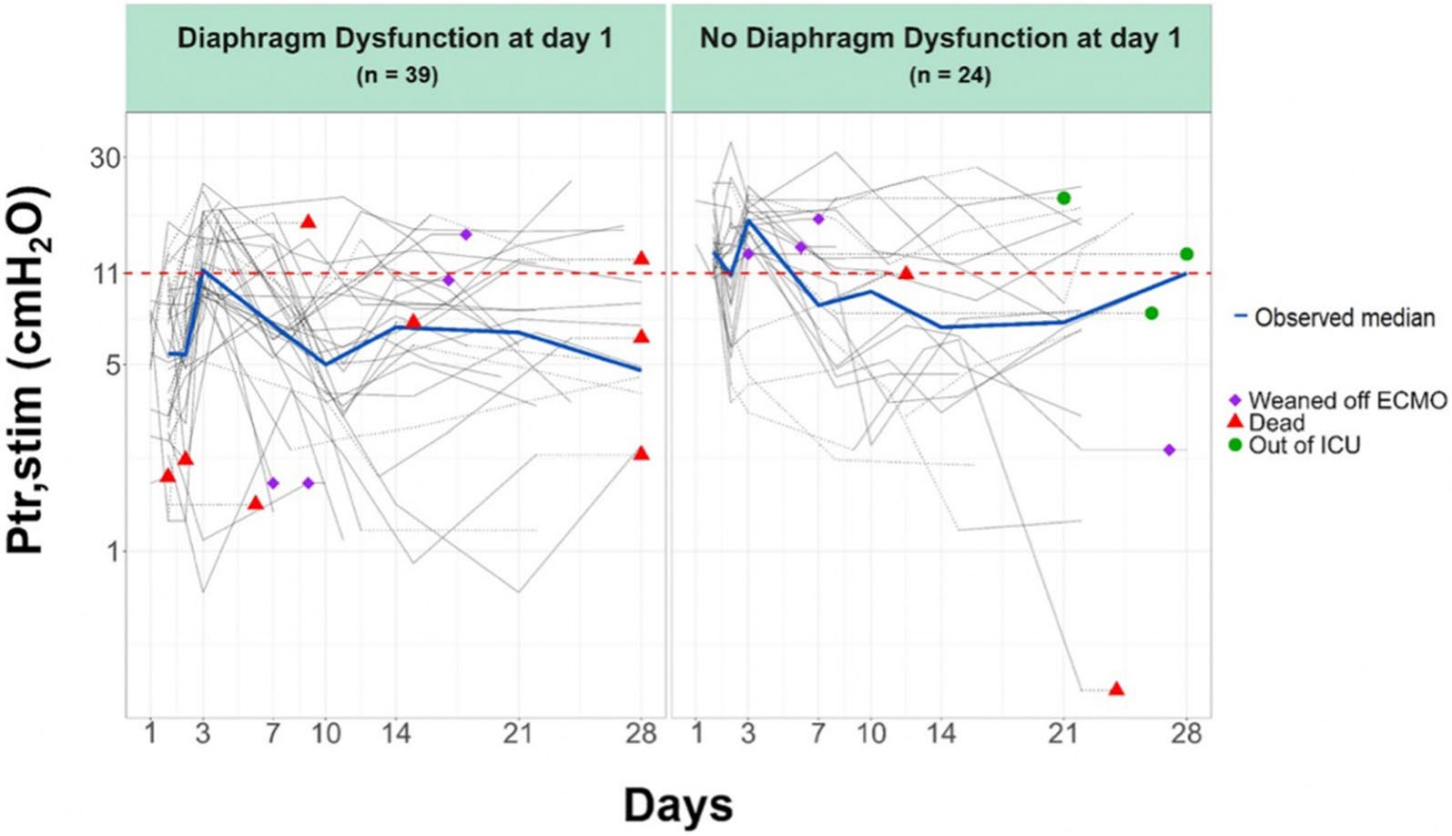
Time course of diaphragm function (logarithm scale) over the ICU course according to the presence or absence of diaphragm dysfunction at ECMO day 1

Ptr,stim was measured at ECMO days 1, 2, and 3 in 63, 51, and 47 patients, respectively. The numbers of patients at the time of the following assessments from D7 to D28 and at the time of ECMO weaning are presented in the online supplement (see Additional file 1: Table SDC-S1). The evolution of the diaphragm function over time was characterized by a large interindividual variability (Fig. 3). In the whole population, between the first (n = 63) and the last available (n = 12) diaphragm function assessment, Ptr,stim did not significantly change (from 8.4 (5.1–12.4) to 8.5 (5.1–13.2) cmH_2_O) (Fig. 3). Among the group of patients with diaphragm dysfunction at day 1, (n = 39), 21 had a Ptr, stim < 11cmH_2_O, 7 a Ptr, Stim ≥ 11cmH_2_O, 5 has not been evaluated, 4 were dead and 2 were weaned from ECMO on day 7. On the other hand, among patients without diaphragm dysfunction at day 1 (n = 24) 12 have Ptr,stim < 11cmH2O, 7 patients a Ptr, Stim ≥ 11cmH2O, 3 has not been evaluated, 1 was dead and 1 was weaned from ECMO on day 7.

### Diaphragm activity evolution

The evolution of diaphragm activity as estimated by the percentage of spontaneous breathing ventilation and Tfdi during the ECMO run is provided in the online supplement (see Additional file 1: Table SDC-S1b). From Day 1 to Day 7, the proportion of spontaneous breathing ventilation and Tfdi were low (see Additional file 1: Table SDC-S2). Linear regression did not identify a significant association between Ptr,stim on Day 7, and the mean percentage of spontaneous breathing ventilation between day 1 and day 6 (*p* = 0.680, see Additional file 1: Table SDC-2). Similarly, no correlation was found between Ptr,stim on Day 7, and the cumulative diaphragm thickening fraction since Day 1 (*p* = 0.698, see Additional file 1: Table SDC-S3). Lastly, % of spontaneous breathing did not differ between patients with Ptr,stim improvement at day 3 compared to those who did not (Additional file 1: Table SDC-S4).

### Clinical outcomes

Among the 63 patients enrolled in the study, 24 (38%) were still alive on Day 60. One patient died after ECMO removal, and the 23 others were successfully separated from mechanical ventilation. Overall, the total duration of mechanical ventilation was 50 (37–71) days. While the differences were not significant, patients without diaphragm dysfunction on Day 1 had a shorter total duration of mechanical ventilation as compared to their counterparts (Table 3). Likewise, the duration of post-ECMO mechanical ventilation was shorter, but non-significantly so, in patients without diaphragm dysfunction on Day 1 as compared to their counterparts as well as the length of stay in the intensive care unit (Table 3). The number of ventilator-acquired pneumonia, length of stay in the ICU, and mortality were not different in patients with or without diaphragm dysfunction on Day 1. Similarly, no differences were reported according to the improvement or worsening of Ptr, stim on day 3 on ECMO (Additional file 1: Table SDC-S4).

**Table 3:** Outcomes according to the presence of diaphragm dysfunction at ECMO Day 1

	All patients (n = 63)	Diaphragm dysfunction (n = 39)	No diaphragm dysfunction (n = 24)	*P* value
Tracheostomy, n (%)	25 (43)	15 (41)	10 (48)	0.600
Number of VAP episodes	3 (2–4)	3 (2–5)	3 (2–4)	0.397
Total MV duration, days	50 (37–71)	50 (34–70)	49 (38–73)	0.845
In survivors (n = 24), days	60 (38–85)	67 (47–82)	42 (35–85)	0.590
MV post-ECMO, days	20 (12–29)	27 (14–31)	14.5 (10.3–23)	0.157
In survivors (n = 24), days	18 (12–29)	27 (14–31)	14 (9.5–21)	0.124
ECMO duration, days	38 (18–56)	36 (26–53)	41 (18–57)	0.705
In survivors (n = 24), days	32 (17–47)	36 (18–45)	27 (14–51)	0.543
ICU length of stay, days	47 (1–83)	61 (11–97)	38 (1–54)	0.298
In survivors, days	65 (46–101)	86 (61–113)	47 (40–89)	0.134
ICU Mortality, n (%)	39 (62)	26 (67)	13 (54)	0.321

*MV* Mechanical ventilation, *VAP* Ventilator acquired pneumonia, *ICU* Intensive care unit

## Discussion

This study reporting on the diaphragm function, characteristics, and outcomes of 63 patients who received ECMO for severe ARDS shows that (1) 39 (62%) of this population had a diaphragm dysfunction on ECMO Day (1; 2) there was no association between diaphragm dysfunction on day 7 and the cumulative percentage of spontaneous breathing within the 1 week of ECMO and (3) diaphragm dysfunction was not associated with any clinical outcomes.

Diaphragm dysfunction is a serious condition frequently encountered in critically ill patients exposed to invasive mechanical ventilation [12, 25]. Diaphragm dysfunction was present in 62% of our patients which is in line with previous studies [12, 25] using magnetic stimulation of the phrenic nerves [14]. However, the severity of diaphragm dysfunction in our patients (Ptr,stim 5.7 cmH_2_O) seemed worse as compared to the studies of Dres et al*.* (Ptr,stim 6.4 cmH_2_O) and Demoule et al*.* (Ptr,stim 6.3 cmH_2_O) [12, 14]. While multiorgan failure and sepsis have been previously reported as risk factors for diaphragm dysfunction [12, 26] this was not the case with our study. This could be explained by the homogeneity of our population with severe ARDS related to the same disease (i.e., SARS CoV-2 infection) with rare extrapulmonary organ dysfunction. Remarkably, the duration of invasive and non-invasive mechanical ventilation before Day 1 was not different between patients with and without diaphragm dysfunction on Day 1. These findings were unexpected since a previous study showed that both, low and excessive diaphragm activity induces diaphragm atrophy (but not necessarily diaphragm dysfunction) [17]. This suggests that neither respiratory muscles under assistance (before intubation under non-invasive oxygenation supports) nor over assistance (after intubation with controlled mechanical ventilation) could have influenced the diaphragm function in this ECMO population.

The level of PEEP before ECMO was significantly higher in patients with diaphragm dysfunction (13 cmH_2_O versus 11.5 cmH_2_O) which is in line with previous findings showing that PEEP causes changes in diaphragm geometry, especially muscle shortening, and decreases in vivo diaphragm contractile function [27, 28]. By displacing the diaphragm in the caudal direction and reducing the length of fibers, mechanical ventilation with high PEEP may induce longitudinal atrophy and subsequent diaphragm dysfunction [27]. If confirmed in further clinical studies, our findings could suggest that high PEEP level might be a risk factor for diaphragm dysfunction which may have important consequences when implementing diaphragm and lung protective ventilation strategies [14, 17]. We also found that the lung ultrasound score at D1 was higher in the group with diaphragm dysfunction (26 (24–28) vs. 24.5 (22–26) *p* = 0.008). These data suggest that the most severe parenchymal damage might be more likely associated with diaphragm dysfunction by exerting a mechanical constraint on the diaphragm geometry. This is a well-known mechanism already described in several physiological studies [28, 29]. In addition, the inflammatory component of the injured lung characterized by edema could be responsible for direct contiguous muscle damage. However, further investigations are still needed to confirm these hypotheses.

While diaphragm dysfunction has been associated with poor prognosis in several studies [12, 13], this association was not straightforward in our population. It could be explained by the very long duration of mechanical ventilation and ICU stay in this population in whom ICU survival is driven by many other potential contributors*.* Another possible explanation is the limited sample size of our ECMO population which precluded reaching statistical significance despite trends toward a worse prognosis for patients who had a diaphragm dysfunction with notably, a longer duration of mechanical ventilation, a longer duration of MV after ECMO weaning and ICU stay. While it does not imply causality, the association between diaphragm dysfunction and prolonged weaning seems consistent with previous data [13].

Our study was designed to serially measure the diaphragm function over time and to explore the role of diaphragm activity in its evolution. For this purpose, the proportion of spontaneous breathing ventilation and the diaphragm thickening fraction were used as surrogates of diaphragm activity. The evolution of the diaphragm function seems to remain stable over time for patients with and without diaphragm dysfunction on Day 1. It contrasts with previous studies reporting a time-dependent decline in diaphragm function with, however, limited sample sizes (< 10 patients) and a short follow-up. One can argue that ECMO might prevent the decline of diaphragm function over time and that ventilatory-induced diaphragm injury occurs before ECMO starts. The second important result is the lack of statistical correlation between the diaphragm activity and diaphragm function. A previous study reported that the inspiratory effort can modulate the diaphragm thickness [13], but the diaphragm function per se was not evaluated [13].

This study has several strengths. First, the population is remarkably homogeneous in terms of baseline characteristics, etiology of ARDS and ECMO, and ventilatory management which provides good external validity in a similar context. Second, we used the reference technique to measure the diaphragm function in mechanically ventilated patients. Third, we performed serial measurements of the diaphragm function with a standardized timing which allows a granular description of its evolution. Last, we performed the study in two centers inside a large academic hospital. This study has also some limitations. First, our study was conducted on COVID-19-related ARDS which may limit the generalizability of our results in non-COVID-19 ARDS on ECMO. Second, because of ICU beds constraint during the peak of the pandemic, the follow-up of the diaphragm function until extubation was difficult as patients were frequently re-transferred to their initial ICU after ECMO weaning. Third, the capacity of the diaphragm to generate pressure is influenced by the volume of the lungs and the lung volume likely increased as the patients recovered. Therefore, the conditions of measurement of the diaphragm function may have changed over the study which may have influenced Ptr,stim measurements. Fourth, we did not study diaphragmatic function in a control group (i.e., patients with COVID-19 on mechanical ventilation without ECMO). Fifth, our study has a limited sample size which challenges the generalization of our findings. Therefore, the impact on outcomes of the diaphragm function in patients on VV-ECMO still warrants further investigation. Because the role of COVID-19 itself with frequent isolated pulmonary dysfunction may explain our findings, further studies are needed in patients with non-COVID-related ARDS on ECMO.

## Conclusion

Nearly two-thirds of patients undergoing ECMO for severe COVID-19-related ARDS had a severe diaphragm dysfunction on ECMO day 1. However, the diaphragmatic function remained stable over time and did not seem to be associated with any outcomes. Furthermore, there was no association between the presence of diaphragm dysfunction on Day 7 and the percentage of spontaneous breathing during the 1 week of ECMO.

### Supplementary Information

**Additional file1: Table SDC-S1.** **a** Evolution of diaphragm function over time from D1 to weaning; **b** Evolution of diaphragm function and diaphragm activity as estimated by the percentage of spontaneous breathing ventilation and diaphragm thickening fraction. **Table SDC-2.** Impact of the cumulative percentage of spontaneous breathing ventilation on the diaphragm function on day 7. **Table SDC-S3.** Impact of the cumulative diaphragm thickening fraction on the diaphragm function at day 7. **Table SDC-S4.** Characteristics, pre ECMO management, spontaneous breathing and outcomes according to improvement or no improvement of Ptr, Stim between day 1 and day 3 on ECMO.

## Data Availability

The datasets analyzed during the current study are available from the corresponding author upon reasonable request.

## Abbreviations

ARDS: Acute respiratory distress syndrome
COVID-19: Coronavirus disease 2019
ECMO: Extracorporeal membrane oxygenation
FiO2: Fraction of inspired oxygen
IBW: Ideal body weight
ICU: Intensive care unit
MV: Mechanical ventilation
PaO2: Partial pressure of alveolar oxygen
PBW: Predicted body weight
PEEP: Positive end-expiratory pressure
SAPS II: Simplified Acute Physiology Score II
SARS-CoV-2: Severe acute respiratory distress syndrome coronavirus 2
SOFA: Sequential Organ Failure Assessment
SpO2: Oxygen saturation measured by pulse oximetry
