# Supplementary material for: Diaphragm function in patients with Covid-19-related acute respiratory distress syndrome on venovenous extracorporeal membrane oxygenation
Source: Ann Intensive Care. 2023 Sep 26;13:92. doi: 10.1186/s13613-023-01179-w (PMC10522552; doi:10.1186/s13613-023-01179-w)
Supplement: Supplementary file 1 — Additional file1: Table SDC-S1. a Evolution of diaphragm function over time from D1 to weaning; b Evolution of diaphragm function and diaphragm activity as estimated by the percentage of spontaneous breathing ventilation and diaphragm thickening fraction. Table SDC-2. Impact of the cumulative percentage of spontaneous breathing ventilation on the diaphragm function on day 7. Table SDC-S3. Impact of the cumulative diaphragm thickening fraction on the diaphragm function at day 7. Table SDC-S4. Characteristics, pre ECMO management, spontaneous breathing and outcomes according to improvement or no improvement of Ptr, Stim between day 1 and day 3 on ECMO. [file 13613_2023_1179_MOESM1_ESM.doc]

**Supplemental Digital Content**

**Diaphragm function in patients with ARDS on venovenous ECMO: data from a COVID-19 cohort**

Melchior Gautier^1,2,3^, Vincent Joussellin^3,4,5^, Jacques Ropers^6^, Lina El Houari^6^, Alexandre Demoule^3,4,5^, Thomas Similowski^3,4,5^, Alain Combes^1,2,3^, Matthieu Schmidt^1,2,3^, Martin Dres^3,4,5^

^1^Sorbonne Université, Institute of Cardiometabolism and Nutrition, Institut National de la Santé et de la Recherche Médicale (INSERM) Unité Mixte de Recherche (UMRS) 1166

^2^Service de Médecine Intensive–Réanimation, Institut de Cardiologie, Assistance Publique–Hôpitaux de Paris (APHP), Hôpital Pitié–Salpêtrière, Paris, France

^3^ Groupe de Recherche Clinique 30 RESPIRE

^4^Sorbonne Université, INSERM, UMRS1158 *Neurophysiologie Respiratoire Expérimentale et Clinique*, F-75005 Paris, France

^5^AP-HP, Groupe Hospitalier Universitaire APHP-Sorbonne Université, hôpital Pitié-Salpêtrière, Département R3S *(Respiration, Réanimation, Réadaptation respiratoire, Sommeil)*, F-75013 Paris, France

^6^ AP-HP, Département de Santé Publique, Assistance Publique–Hôpitaux de Paris (APHP), Hôpital Pitié–Salpêtrière, Paris, France

**Table SDC-1.** a) Evolution of diaphragm function over time from D1 to weaning; b) Evolution of diaphragm function and diaphragm activity as estimated by the percentage of spontaneous breathing ventilation and diaphragm thickening fraction

| **Days** | **Day 1** | **Day 2** | **Day 3** | **Day 7** | **Day 10** | **Day 14** | **Day 21** | **Day 28** | **Off ECMO** |
| --- | --- | --- | --- | --- | --- | --- | --- | --- | --- |
| **Ptr,Stim**  **cmH_2_O** | 8.4  [4.9-12.5] | 8  [5.0-12.5] | 15.7  [9.9-18.9] | 7.5  [5.7-12.0] | 6.3  [3.6-11.2] | 7.2  [5.1-10.6] | 6.9  [3.6-12.2] | 7.75  [4.5-12.7] | 8.5  [5.1-13.2] |
| **n** | 63 | 51 | 47 | 47 | 42 | 39 | 35 | 30 | 12 |

**a.**

b.

|  | **Day 1** | **Day 2** | **Day 3** | **Day 7** |
| --- | --- | --- | --- | --- |
| **Ptr,stim, cmH_2_O** | 8.4 [4.9-12.5] | 8 [5-12.55] | 15.7 [9.85-18.85] | 7.5 [5.65-12] |
| **Spontaneous breathing ventilation, %** | 1.57 [0.2-8.35] | 2.85 [0.83-10] | 4.3 [1-18.25] | 9.1[3.66-20] |
| **Diaphragm thickening fraction, %** | 6.2 [0.0-7.0] | 6.8 [0.0-8.0] | 7.4 [0.0-9.0] | 6.7 [0.0-8.0] |

Ptr,stim: tracheal pressure generated by bilateral anterior magnetic phrenic nerve stimulation

**Table SDC-2.** Impact of the cumulative percentage of spontaneous breathing ventilation on the diaphragm function on day 7

|  | **Parameter estimate** | **CI Lower bound (95%)** | **CI Upper bound (95%)** | **P value** |
| --- | --- | --- | --- | --- |
| **Intercept** | 10.449 | 3.574 | 17.316 | 0.009 |
| **Cumulative percentage of spontaneous breathing ventilation** | -0.016 | -0.093 | 0.060 | 0.680 |

**Table SDC-3.** Impact of the cumulative diaphragm thickening fraction on the diaphragm function at day 7

|  | **Parameter estimate** | **CI Lower bound (95%)** | **CI Upper bound (95%)** | **P value** |
| --- | --- | --- | --- | --- |
| **Intercept** | 9.312 | 2.426 | 16.197 | 0.016 |
| **Cumulative diaphragm thickening fraction** | 1.992 | -7.906 | 11.889 | 0.698 |

**Table SDC-4.** Characteristics, pre ECMO management, spontaneous breathing and outcomes according to improvement or no improvement of Ptr, Stim between day 1 and day 3 on ECMO

|  |  | **Between Day 1 and Day 3 on ECMO** | |  |
| --- | --- | --- | --- | --- |
| **Variables** | **All patients**  **(n=63)** | **Ptr,Stim improvement**  **(n=41)*** | **No improvement of the Ptr, Stim**  **(n=17)**** | ***P* value** |
| Age, years, median (IQR) | 53 (42 – 59) | 50 (40,5 – 58,5) | 54 (43 – 59) | 0.603 |
| Female sex, n (%) | 16 (25) | 11 (26) | 5 (29) | 0.583 |
| Body mass index, kg/m^2^, median (IQR) | 33 (29 – 37) | 32 (29 – 35,5) | 34 (27 – 38) | 0. 583 |
| SAPS II, median (IQR) | 56 (45 – 66) | 56 (44 – 66) | 56 (46 – 72) | 0.262 |
| SOFA, median (IQR) | 12 (9 – 12) | 12 (9 –12) | 12 (9 – 13) | 0.357 |
| Charlson ≥ 1, n (%) | 25 (40) | 10 (24) | 6 (35) | 0.750 |
| **Pre ECMO management** |  |  |  |  |
| High Flow Nasal Oxygen, n (%) | 53 (85) | 38 (92) | 14 (82) | 0.345 |
| Duration, days, median (IQR) | 5 (2 – 7) | 5 (1 – 7) | 5 (3 – 7) | 0.990 |
| Non-invasive ventilation, n (%) | 33 (54) | 23 (56) | 10 (58) | 0.999 |
| Duration, days, median (IQR) | 6 (2 – 7) | 6 (2 - 8) | 5 (2 – 7) | 0.335 |
| Duration of MV before ECMO, median (IQR) | 4 (3 – 6) | 4 (3 – 7) | 4 (1 – 5) | 0.050 |
| **Pre-ECMO ventilator settings** |  |  |  |  |
| Tidal volume, ml/kg PBW, median (IQR) | 6.0 (5.5 – 6.3) | 6.0 (5.6 – 6.3) | 6.0 (5.2 – 6.4) | 0.656 |
| Respiratory rate, min^-1^, median (IQR) | 30 (30 – 33) | 30 (30 – 33) | 32 (29 – 35) | 0.408 |
| Driving pressure, cmH_2_O, median (IQR) | 19 (16 – 21) | 19 ( 16 – 22) | 19 (20 – 24) | 0.125 |
| Positive end-expiratory pressure, cmH_2_O, median (IQR) | 12 (10 – 14) | 12 (10 – 15) | 12 (9 – 14) | 0.274 |
| Respiratory system compliance, mL/cmH_2_O, median (IQR) | 22 (16 – 25) | 22 (16 – 26) | 21 (15 – 24) | 0.708 |
| **% Spontaneous breathing within the first 3 days on ECMO** | 5 (1 – 12) | 5 (2 – 11) | 8.1 (1 – 14) | 0.652 |
| **Outcomes** |  |  |  |  |
| Number of VAP episodes | 3 (2 – 4) | 3 (2 – 4) | 3 (2 – 4) | 0.245 |
| Total MV duration, days | 50 (37 – 71) | 74 (52 – 96) | 38 (26 – 86) | 0.810 |
| MV post-ECMO, days | 20 (12 – 29) | 24 (14 – 35) | 12 (8 – 25) | 0.08 |
| ECMO duration, days | 38 (18 – 56) | 36 (18 – 57) | 18 (10 – 38) | 0.203 |
| ICU length of stay, days | 47 (1 – 83) | 65 (39 – 101) | 46 (17 – 84) | 0.396 |
| **In survivors** | n=24 | n=15 | n=9 |  |
| Total MV duration, days | 60 (38 – 85) | 71 (47 – 93) | 38 (26­ – 85) | 0.109 |
| MV post ECMO, days | 18 (12 – 29) | 22 (14 – 31) | 8 (12 – 25) | 0.188 |
| ECMO duration, days | 32 (17 – 47) | 36 (18 – 57) | 18 (10 – 38) | 0.153 |
| ICU length of stay, days | 65 (46 – 101) | 86 (55 – 103) | 38 (26 – 85) | 0.276 |
| ICU Mortality, n (%) | 39 (62) | 27(65) | 8 (47) | 0.241 |

*Ptr,Stim improvement was defined as Ptr, stim (J3) - Ptr, stim (J1)>0 whereas no improvement of Ptr,Stim was defined as Ptr, stim (J3) - Ptr, stim (J1)<0*

**Missing data in 3 patients*

***Missing data in 2 patients*
